# Elimination of Self-Reactive T Cells in the Thymus: A Timeline for Negative Selection

**DOI:** 10.1371/journal.pbio.1001566

**Published:** 2013-05-21

**Authors:** Ivan Lilyanov Dzhagalov, Katherine Grace Chen, Paul Herzmark, Ellen A. Robey

**Affiliations:** Division of Immunology and Pathogenesis, Department of Molecular and Cell Biology, University of California, Berkeley, California, United States of America; University of Pennsylvania, United States of America

## Abstract

The elimination of autoreactive T cells occurs via thymocyte apoptosis and removal by thymic phagocytes, but the sequence of events *in vivo*, and the relationship between thymocyte death and phagocytic clearance, are unknown. Here we address these questions by following a synchronized cohort of thymocytes undergoing negative selection within a three-dimensional thymic tissue environment, from the initial encounter with a negative selecting ligand to thymocyte death and clearance. Encounter with cognate peptide–MHC complexes results in rapid calcium flux and migratory arrest in auto-reactive thymocytes over a broad range of peptide concentrations, followed by a lag period in which gene expression changes occurred, but there was little sign of thymocyte death. Caspase 3 activation and thymocyte loss were first detectable at 2 and 3 hours, respectively, and entry of individual thymocytes into the death program occurred asynchronously over the next 10 hours. Two-photon time-lapse imaging revealed that thymocyte death and phagocytosis occurred simultaneously, often with thymocytes engulfed prior to changes in chromatin and membrane permeability. Our data provide a timeline for negative selection and reveal close coupling between cell death and clearance in the thymus.

## Abstract 2

Two-photon microscopy and flow cytometry reveal the timing of thymocyte death and the surprisingly close coupling between cell death and phagocytosis during negative selection in thymic slices.

## Author Summary

As an important safeguard against autoimmunity, T cells bearing autoreactive T cell antigen receptors are eliminated during their development in the thymus, a process known as negative selection. Although much is known about the molecular events involved in negative selection, surprisingly little is known about the dynamic aspects of the process. Here we examine a synchronized population of developing T cells (thymocytes) undergoing negative selection within three-dimensional living thymic tissue. We show that the initial encounter with negative selecting ligands results in migratory arrest, but in spite of this synchronous early response, individual thymocytes then undergo delayed and asynchronous entry into the death program between 2 and 12 hours thereafter. Using time-lapse two-photon imaging, we reveal that thymocyte death and the clearance of the dead cells invariably occur together, with many thymocytes already engulfed by a macrophage before the cell death-related changes in chromatin and membrane permeability are evident. These data provide a timeline of the major events during negative selection, and suggest close coupling between thymocyte death and clearance by macrophages.

## Introduction

As an important safeguard against autoimmunity, T cells bearing autoreactive T cell antigen receptors (TCRs) are eliminated during their development in the thymus, a process known as negative selection. Although much is known about the molecular events involved in negative selection [1], surprisingly little is known about the dynamic aspects of the process. For example, what is the sequence of events from the first encounter with a negative selecting ligand until the death of the thymocyte? How long does the process take? Does a single encounter with self-peptide lead to migratory arrest, or do thymocytes remain motile and continue to sample the tissue for negative selecting ligands? What is the temporal relationship between the death of the thymocyte and its clearance by phagocytes?

Pioneering studies carried out in the 1980s documented the absence of thymocyte populations bearing self-reactive TCRs in mice expressing the relevant self antigens [2]–[4]. However, these studies could not address the timing and mechanism of negative selection since self-antigens were present continuously and dying thymocytes were not detected. In subsequent experiments, rearranged TCR transgenes were expressed in mice without the relevant self-antigen, and then antigenic peptides were administered *in vivo* to induce negative selection [5]–[8]. *In vitro* co-cultures of transgenic thymocytes with cognate peptide-loaded antigen-presenting cells (APCs) have also been used to examine the process of negative selection [9]–[11]. These approaches have revealed extensive thymocyte death accompanied by nuclear condensation and other classic signs of programmed cell death, or apoptosis. However, the analyses were typically performed at a single time point, often a day or more after peptide addition, and thus it was unclear when the process of negative selection began and ended. In addition, the impact of systemic cytokines produced by mature T cell stimulation in the periphery was a confounding factor in many of the *in vivo* studies [12],[13]. A recent study examining negative selection to endogenous self-antigen *in vivo*, reported a decrease in the number of autoreactive thymocytes becoming apparent 24–48 hours after the first signs of TCR triggering [14]. These delayed and asynchronous kinetics were attributed to the variable time for a thymocyte to encounter a thymic APC capable of providing a negative selecting signal. Thus, there remains considerable uncertainty regarding the timing of thymocyte death during negative selection.

Another outstanding question is the relationship between thymocyte death and clearance by macrophages *in vivo*. *In vitro* studies of cultured cells undergoing apoptosis indicate that mitochondrial damage and caspase activation are followed by dismantling of cellular components accompanied by nuclear condensation, membrane blebbing, and exposure of phosphatidylserine (PS) on the outer face of the plasma membrane [15]. *In vivo*, PS exposure is thought to serve as an “eat-me” signal to phagocytes, promoting the removal of membrane blebs and apoptotic cells and preventing the release of cellular contents into extracellular space [16]. In the thymus, very few dying cells are observed at steady state, and apoptotic cells are found inside phagocytes, indicating efficient clearance mechanisms [17]. However, it is unclear whether thymocytes first undergo apoptosis and then are rapidly engulfed, or whether the engulfment by macrophages precedes thymocyte death. Determining whether apoptosis precedes phagocytosis or vice versa is important, both for understanding the mechanistic link between these events *in vivo*, and because the occurrence of cell death before phagocytosis may lead to greater potential inflammation due to the release of cellular contents.

Studies of negative selection *in vivo* have largely focused on the end result of thymocyte self-reactivity, and we know little about the initial encounters between autoreactive thymocytes and thymic APCs presenting negative selecting ligands. For mature T cells in lymph nodes, the initial encounters with peptide–MHC-bearing dendritic cells can occur as transient, serial interactions prior to migratory arrest and stable conjugate formation, particularly under conditions of suboptimal stimulation [18]–[20]. An indication that autoreactive thymocytes may also engage in serial contacts with APC during negative selection comes from a steady-state model in which thymocytes undergo negative selection to a tissue-restricted antigen expressed in the medulla [21]. In this system a large number of autoreactive thymocytes persisted and remained motile in the thymic medulla, exhibiting a confined migration pattern that allowed for serial contact with multiple dendritic cells. However, because antigen was present continuously, it was unclear whether confined migration occurred during the initial contact with antigen, or reflected an adaptation of thymocytes to antigen exposure over time. Moreover, this model is based on a specialized form of negative selection in which medullary thymic epithelial cells exhibit stochastic and low-level expression of proteins that are otherwise restricted to peripheral tissues [22],[23]. Much of the negative selection in the thymus is driven by ubiquitous, rather than tissue-restricted, self antigens, and these different forms of negative selection likely differ in terms of the abundance and spatial distribution of antigens, types of peptide-presenting cells, and molecular requirements [14],[22]–[24].

Here we examine a cohort of thymocytes undergoing negative selection to a ubiquitous antigen within three-dimensional living thymic tissue. The initial encounter with negative selecting ligands leads to a rapid rise in intracellular calcium and migratory arrest over a broad range of peptide concentrations. Thymocytes with active caspase 3 are detectable starting at 2 hours after peptide addition, while other indicators of cell death, including changes in chromatin structure and membrane permeability, first become apparent at 3 h. In spite of the synchronous early response to negative selecting ligand, individual thymocytes undergo delayed and asynchronous entry into the death program from 2–12 hours after peptide addition. Time-lapse two-photon imaging revealed that thymocyte death and phagocytosis invariably occur together, with many thymocytes already engulfed by a macrophage before the changes in chromatin and membrane permeability are evident. These data provide a timeline of the major events during negative selection, and suggest close coupling between the thymocyte death and clearance by macrophages.

## Results

### Comparison of Thymocyte Activation and Cell Death during Negative Selection *In Situ* versus *In Vitro*

**Figure 1:**
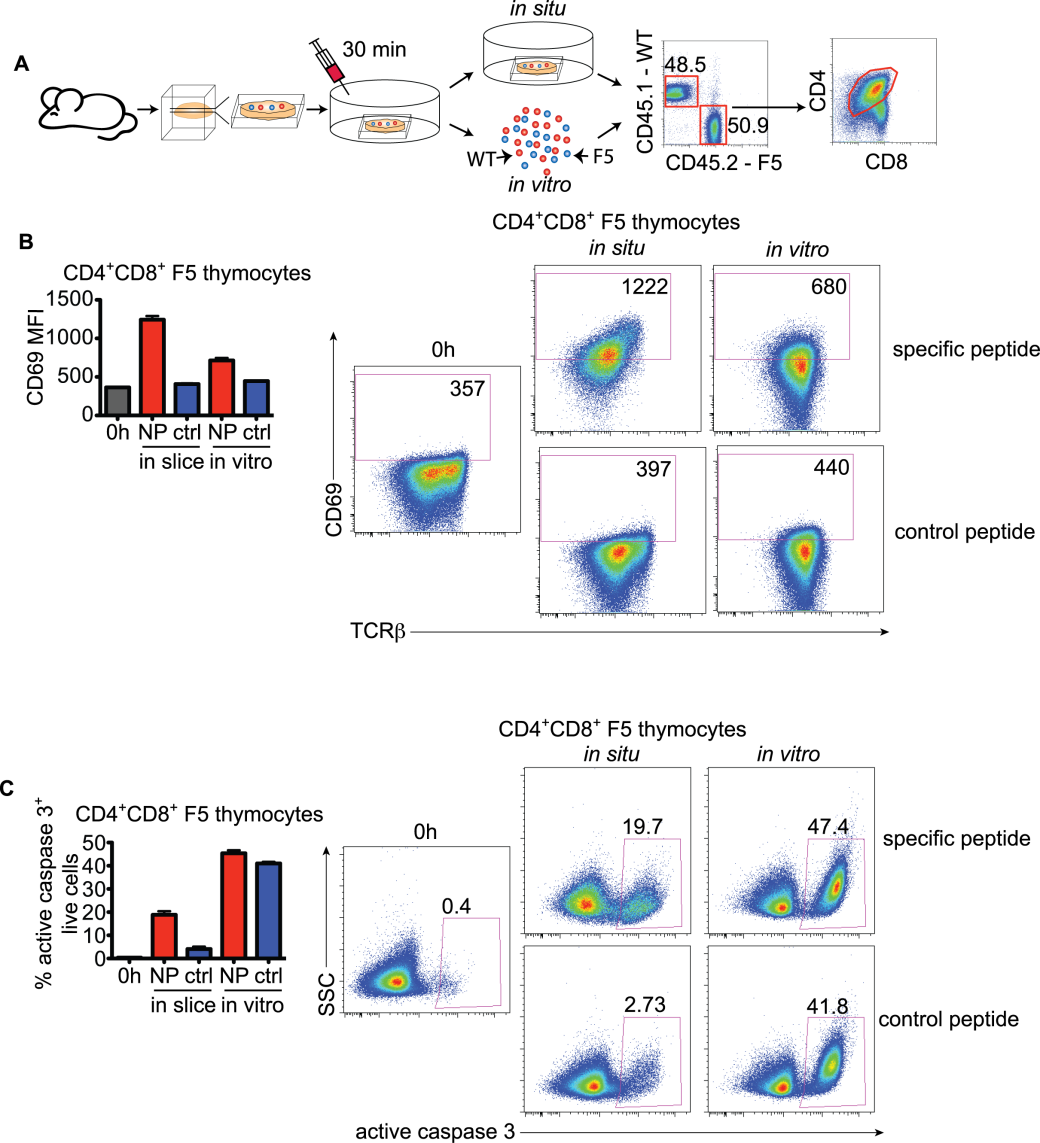
Comparison of thymocyte activation and apoptosis *in vitro* and *in situ*. A) Cartoon of the experimental procedure: Slices from the thymus of a mixed BM chimera mouse that contain allelically marked F5 thymocytes were treated for 30 min with specific or control peptide and then either dissociated in single cell suspension (*in vitro*) or incubated intact as a slice (*in situ*). Ten hours later the samples were analyzed by flow cytometry. Examples of the gating strategy to distinguish F5 and polyclonal cells and the CD4^+^CD8^+^ (DP) population are shown to the right. B) Expression of CD69 on gated F5 CD4^+^CD8^+^ thymocytes after treatment with specific or control peptide and incubation *in situ* or *in vitro*. For comparison, the level of CD69 expression without treatment is shown in grey. Representative flow cytometry plots are shown to the right. The number in each plot is the mean fluorescence intensity (MFI) of CD69 staining. C) Active caspase 3 induction in F5 CD4^+^CD8^+^ thymocytes after treatment with specific or control peptides and incubation *in situ* or *in vitro*. Gated on cells with intact plasma membrane (Aqua^−^). Representative flow cytometry plots are shown to the right. The number in each plot is the percentage of active caspase 3^+^ cells among F5 CD4^+^CD8^+^ thymocytes. The results are expressed as mean with SEM and are representative of three independent experiments done in triplicates or quadriplicates.

The majority of the studies of negative selection have utilized *in vitro* models that do not support thymocytes' normal motility, nor their dynamic interactions with cells in the three-dimensional tissue environment. To examine the impact of these factors on negative selection, we first compared the activation and death of thymocytes in response to negative selection signals in intact three-dimensional versus dissociated tissue. We incubated thymic slices containing F5 TCR transgenic thymocytes for 30 minutes with specific peptide (NP_366–374_ derived from influenza nucleoprotein) to mimic negative selection to a ubiquitous antigen and then continued the incubation for 10 hours either as an intact slice “*in situ*” or after dissociation of the tissue “*in vitro*” (Fig. 1A). We then analyzed thymocytes by flow cytometry for up-regulation of the activation marker CD69 (Fig. 1B) and induction of active caspase 3, an early marker of apoptosis (Fig. 1C). We focused our analysis on CD4^+^CD8^+^ double positive (DP) thymocytes, since this population can be a target of deletion to ubiquitous self-antigens [14]. Because of the down-regulation of CD4 and CD8 following TCR stimulation, a phenomenon known as DP “dulling”, we adjusted the DP gate to include the CD4^low^CD8^low^ population [11],[25]. Interestingly, CD4^+^CD8^+^ F5 thymocytes from intact slices showed greater CD69 up-regulation in response to specific peptide relative to dissociated slices (Fig. 1B). Moreover, F5 thymocytes from intact thymic slices incubated with control peptide showed a lower level of non-specific cell death compared to thymocytes cultured *in vitro*. (Fig. 1C). These results demonstrate that intact thymic slices are superior to dissociated thymic tissue for detection of specific cell death during negative selection, due to both more efficient responses to negative selecting ligands and lower levels of non-specific cell death.

### Rapid and Synchronous Activation of Antigen-Specific Thymocytes in Thymic Slices

**Figure 2:**
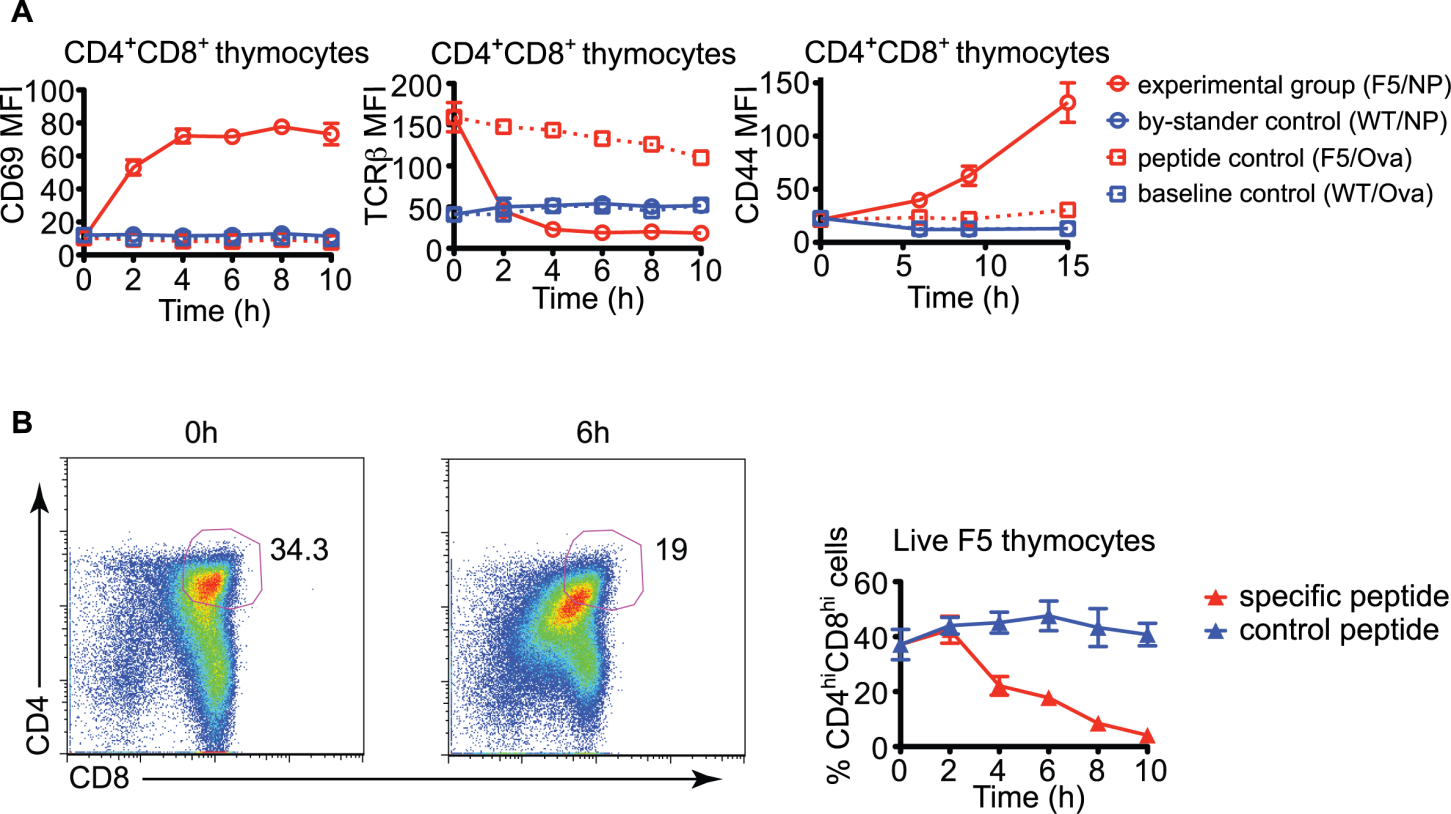
Rapid and synchronous activation of TCR transgenic thymocytes in thymic slices. Thymic slices from mixed BM chimeric mice (F5+WT) were incubated with either specific (NP) or control (Ova) peptides and cultured for various times followed by flow cytometric analysis. A) Changes in the surface expression of CD69, TCRβ and CD44 on gated CD4^+^CD8^+^ thymocytes. B) Changes in the proportion of CD4^hi^CD8^hi^ cells over time among the total thymocytes due to down-regulation of surface CD4 and CD8. Representative flow cytometry plots are shown on the left. The data represents mean with SEM from quadruplicate samples and is from a representative experiment out of at least three independent experiments.

To establish a timeline of cell activation, apoptosis, and clearance during negative selection, we initiated a wave of negative selection by overlaying thymic slices containing F5 TCR transgenic thymocytes with specific peptide. The thymic slices were prepared from lethally-irradiated mice reconstituted with mixtures of wild type (WT) and F5 TCR transgenic bone marrow (BM), allowing us to compare the response of polyclonal thymocytes as an internal control. Up-regulation of CD69 was first detected on CD4^+^CD8^+^ F5 thymocytes one hour after specific peptide addition (data not shown) and peaked between 2 to 4 hours, corresponding with the down-regulation of TCRβ (Fig. 2A). Another activation marker, CD44 was also up-regulated in the experimental group, but with slightly delayed kinetics (Fig. 2A). Finally, the down-regulation of CD4 and CD8, a phenomenon known as DP “dulling” that is indicative of TCR signaling, could be detected starting 4 hours after specific peptide stimulation (Fig. 2B) [11],[25]. No changes in these activation markers were observed on CD4^+^CD8^+^ F5 thymocytes from slices treated with a control peptide or on WT cells alongside activated F5 thymocytes, confirming that the changes were due to direct TCR engagement on F5 thymocytes rather than cytokine-driven bystander effects or non-specific signals. Altogether, these data show that F5 thymocytes are synchronously and specifically activated by the cognate peptide addition to thymic slices, leading to rapid changes in gene expression.

### Antigen-Specific Thymocytes Undergo Rapid Migratory Arrest after Encounter with Cognate Ligand

**Figure 3:**
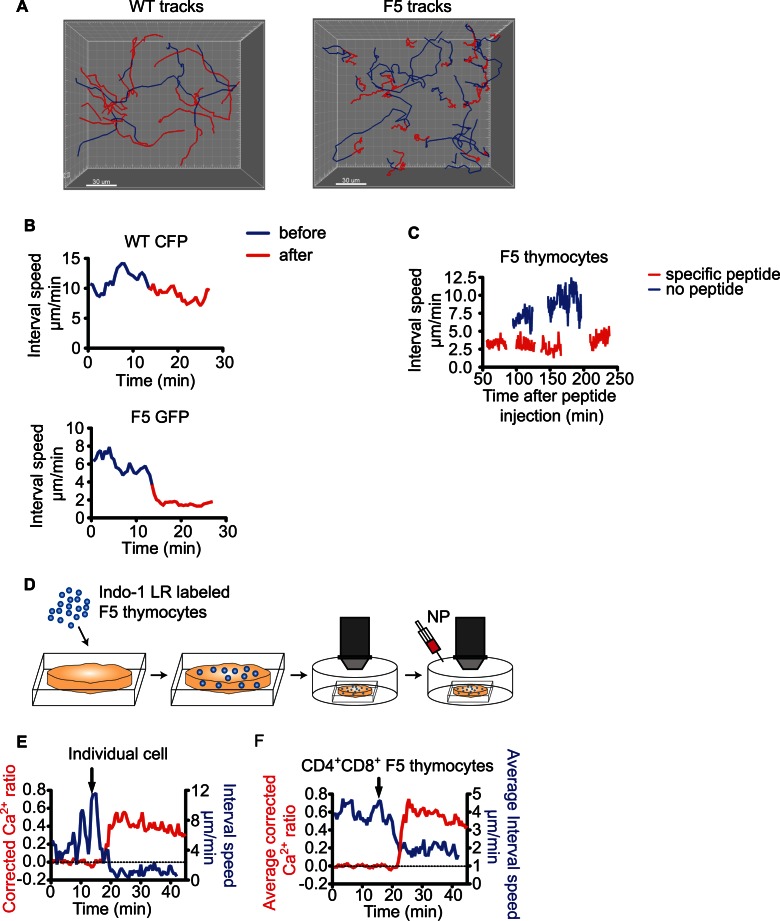
Antigen-specific thymocytes arrest within seconds after encountering their cognate peptide-MHC ligand. Thymus slices from BM chimeras containing GFP-expressing F5 thymocytes and CFP-expressing WT thymocytes were imaged by two-photon microscopy and peptide was added during the imaging run. (A) The trajectories of WT and F5 cells within the same imaging volume are represented as tracks that are color-coded blue (before the addition of the specific peptide) and red (after the addition of the specific peptide). One micromolar NP_366–374_ peptide was added to the perfusion medium 13.5 min after the beginning of the movie. The movie was taken in the cortex. (B) Changes in the interval speed (150 sec) of WT and F5 CD4^+^CD8^+^ thymocytes from (A). The data represent average values for 25 WT and 25 F5 cells. (C) Graph of the average interval speed (30 sec) of cortical (CD4^+^CD8^+^) F5 thymocytes after injection of specific peptide (red) in a mixed BM chimeric mouse. For comparison the average interval speed (30 sec) of cortical F5 cells in the absence of the cognate peptide is shown (blue). Four movies were taken in succession after the injection in different imaging volumes within the same sample. (D) Cartoon of the experimental system for imaging the changes in the intracellular Ca^2+^ concentration: WT thymic slices were overlaid with Indo-1 LR labeled purified CD4^+^CD8^+^ F5 thymocytes isolated from a positive selecting background (H2^b^). Specific peptide was added while time-lapse two-photon microscopy was performed. (E) Changes of the Ca^2+^ concentration expressed as corrected Ca^2+^-ratio and the interval speed (150 sec) of a single cell after addition of 1 nM specific peptide. (F) Changes in the average Ca^2+^ concentration expressed as corrected Ca^2+^-ratio and the average interval speed (150 sec) for all F5 cells in the imaging volume after addition of 1 nM specific peptide. The arrows in (E) and (F) indicate the time of peptide addition.

To visualize the earliest response of thymocytes to their negative selecting ligand, we modified our system to accommodate time-lapse imaging. We used thymic slices from mixed BM chimeric mice in which a small proportion of the thymocytes express the F5 TCR and green fluorescent protein (GFP). WT (polyclonal TCR) thymocytes labeled with cyan fluorescent protein (CFP) served as an internal control. Cortex and medulla were distinguished based on the proximity of the imaging volume to the capsule of the thymus and the characteristically lower position of the medulla (Fig. S1). We then performed two-photon microscopy of thymocytes within cortical regions (corresponding to CD4^+^CD8^+^ thymocytes) and midway though the imaging run, we added specific peptide to the perfusion medium. TCR transgenic thymocytes arrested their migration within minutes of peptide addition (Video S1 and Fig. 3A and 3B). The WT thymocytes in the same imaging volume did not alter their migration pattern confirming that the stopping was induced directly by TCR triggering, rather than by alterations in the tissue microenvironment. The F5 thymocytes maintained low speed for hours after the encounter with their cognate pMHC ligand (Fig. 3C). Migratory arrest was also observed when the experiment was performed with CD4^+^CD8^+^ F5 thymocytes purified by depletion of CD8SP and CD4^−^CD8^−^ populations and overlaid onto thymic slices with OT I thymocytes serving as controls (Video S2) [26].

To examine the relationship between thymocyte behavior and TCR signaling, we adapted the experimental system to allow for measurement of TCR induced calcium flux using the fluorescent Ca^2+^ indicator dye Indo-1 LR (Fig. 3D). We isolated CD4^+^CD8^+^ thymocytes from F5 TCR transgenic mice, loaded them with Indo-1 LR, and overlaid them onto thymic slices. Labeled thymocytes were found in both the cortex and medulla of thymic slices (Fig. S1). This likely reflects the physiological trafficking of post-selection thymocytes in our samples, which are in the process of becoming CD8^+^CD4^−^ single positive (CD8SP) and are beginning to express homing molecules, such as CCR7, that give them access to the medulla [27],[28]. In contrast, “pre-selection” CD4^+^CD8^+^, isolated from non-selecting backgrounds localize overwhelmingly to the cortex [29] (our unpublished observations) consistent with their less mature phenotype. We performed all subsequent imaging studies in the medullary region due to the higher cell densities and superior image quality compared to the cortex.

CD4^+^CD8^+^ thymocytes exhibited a relatively low intracellular calcium concentration and high motility in thymic slices prior to peptide addition, whereas addition of cognate peptide triggered a sharp increase in the intracellular Ca^2+^ concentration and a sudden drop in thymocyte motility. These effects could be observed in individual thymocytes (Video S3 and Fig. 3E) and at the population level (Fig. 3F). The data confirm the inverse relationship between thymocyte motility and calcium signaling previously observed [26] and indicate that peptide diffusion in the tissue, loading onto MHC molecules, and encounter of thymocytes with peptide-MHC bearing cells occur rapidly in this system.

### Rapid Ca^2+^ Flux and Migratory Arrest Occur over a Wide Range of Peptide Concentrations

**Figure 4:**
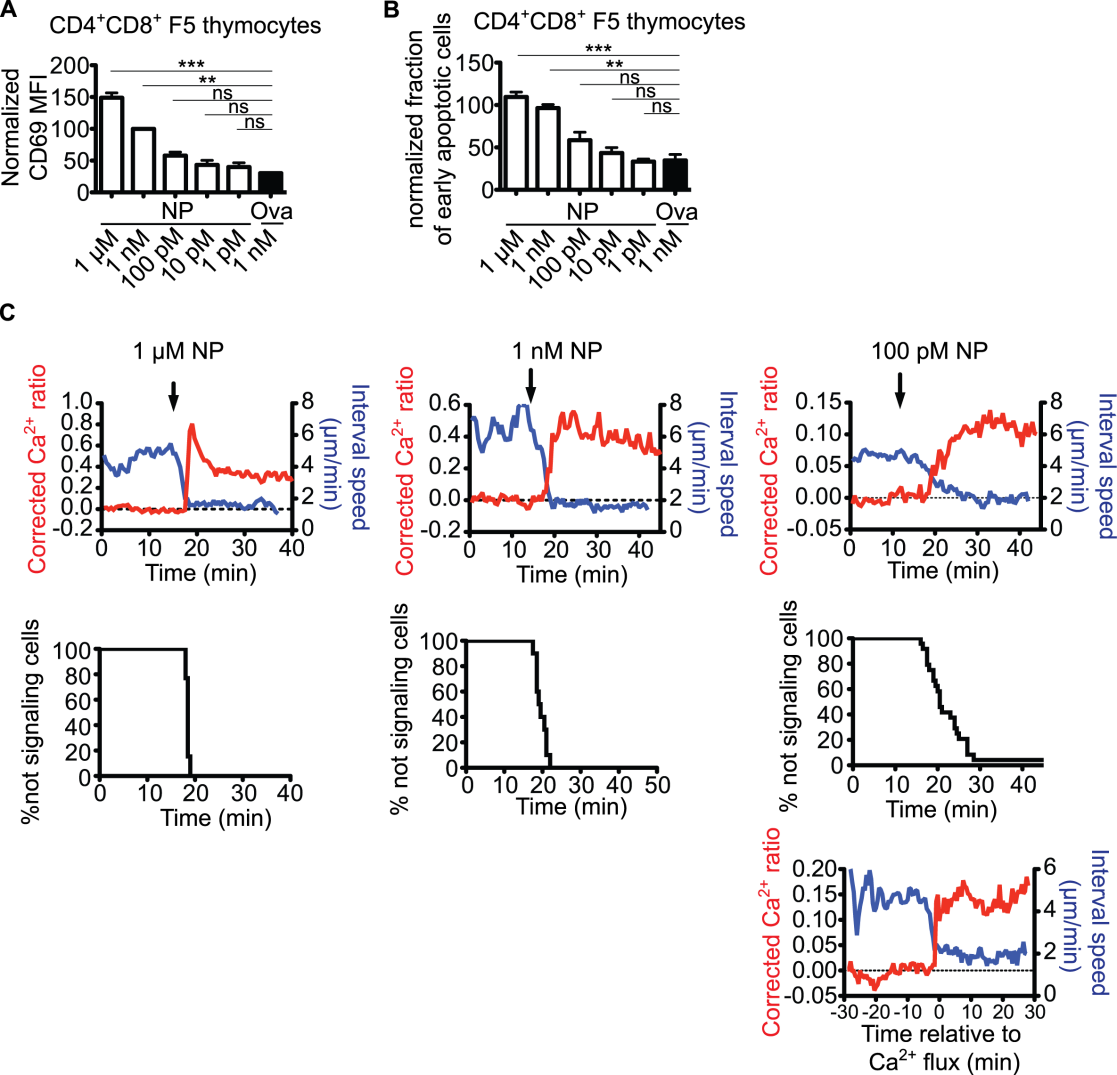
Rapid calcium changes and migratory arrest over a wide range of peptide concentrations. (A) Changes in the expression of CD69 after stimulation with different doses of specific or control peptide. (B) Changes in the proportions of early apoptotic cells (active caspase 3^+^ Aqua^−^) after stimulation with different doses of specific or control peptide. Data in (A) and (B) represent compilations from two to four individual experiments per concentration done in quadriplicates. To normalize the values, 1 nM specific peptide (NP) was set to 100%. (C) Top row: Relationship between motility (average interval speed over 150 seconds) and calcium concentration (Ca^2+^-bound/Ca^2+^-free ratio) after treatment of Indo-1 LR-labeled purified CD4^+^CD8^+^ F5 overlaid on thymic slices with different concentrations of specific peptide. Average values for all tracked cells (n = 21 for 1 µM, n = 25 for 1 nM and n = 52 for 100 pM) at each time point of the run are shown. Arrows indicate time of peptide addition. Dotted line indicates the average calcium ratio before peptide addition that is set to zero. Below are plots of the proportion of cells responding to the peptide stimulation over time determined by calcium flux and stopping. Only tracks that last >90% of the imaging time were used for the analysis (n = 9 for 1 µM, n = 13 for 1 nM and n = 24 for 100 pM). For the sample with 100 pM specific peptide, the bottom plot shows the same tracks aligned based on the beginning of the calcium signal, and the average calcium changes and motility changes are displayed relative to the start of the signal.

The rapid migratory arrest observed for thymocytes encountering a negative selecting ligand resembles the behavior of mature T cells upon encountering an antigen-bearing dendritic cell (DC) under conditions of optimal priming [18]. On the other hand, less intense stimuli, including low peptide concentration, can lead to an initial phase of transient interactions of mature T cells with DC before forming stable contacts [19]. To determine whether limiting peptide concentration could also lead to transient interactions of thymocytes during negative selection, we first determined the minimal peptide concentrations that induce efficient negative selection in our system. We incubated thymic slices with different concentrations of NP peptide for 10 hours, and examined CD69 and active caspase 3 on F5 thymocytes by flow cytometry. Addition of 1 µM NP peptide induced strong CD69 and active caspase 3^+^ up-regulation, whereas 1 nM led to weaker but significant up-regulation (Fig. 4A and B). Addition of 100 pM NP led to only slightly increased activation and apoptosis induction compared to control peptide, a difference that did not reach statistical significance.

We then tested this range of peptide concentrations for their ability to induce calcium flux and migratory arrest of F5 thymocytes. Interestingly, all peptide concentrations tested induced Ca^2+^ flux and migratory arrest in the vast majority of thymocytes within 20 minutes of peptide addition (Fig. 4C and Video S4). Limiting peptide concentration affected the time required for the majority of thymocytes to respond: within a minute for the highest concentration tested (1 µM), 3–4 min with 1 nM and 15–20 min with 100 pM. In spite of this delay at the population level, individual thymocytes exposed to low peptide concentrations converted rapidly from non-signaling to signaling behavior (Video S4), suggesting that the delay in response at the population level was due to the lower probability of encountering APCs displaying sufficient number of pMHC to trigger a response. To confirm this, we aligned individual cell tracks based on the time point at which elevated calcium was first detected, and calculated the average calcium ratio and interval speed relative to the onset of signaling (Fig. 4C). This analysis confirmed that low peptide concentrations induced an all-or-nothing response in thymocytes, with calcium influx and stopping occurring together, and reaching a maximum over a period of less than 30 sec (Fig. 4C). These data indicate that thymocytes that encounter even a low concentration of negative selecting peptide undergo rapid TCR triggering and migratory arrest.

### Delayed and Asynchronous Entry of Individual Thymocytes into the Death Program

**Figure 5:**
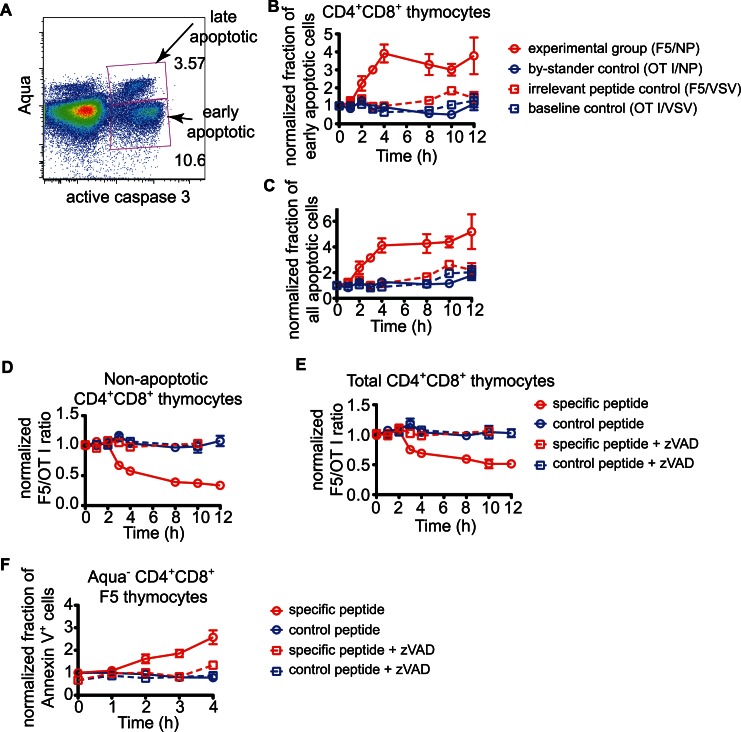
Delayed and asynchronous cell death in response to negative selecting peptide. (A) Example of the gating strategy used to distinguish early apoptotic from late apoptotic cells using staining for active caspase 3 and fixable live/dead dye Aqua. (B) Kinetics of detection of early apoptotic cells (active caspase 3^+^Aqua^−^) in CD4^+^CD8^+^ F5 thymocytes. (C) Kinetics of detection of apoptotic cells (active caspase 3^+^Aqua^+^ or ^−^) in CD4^+^CD8^+^ F5 thymocytes. The data in (B) and (C) depict mean with SEM compiled from five experiments done in triplicates and are normalized so that the proportion of caspase 3^+^ cells at time zero is set to one for each experiment and cell type. D) Ratio of non-apoptotic (active caspase 3^−^Aqua^−^) CD4^+^CD8^+^ F5 and OT I thymocytes recovered from thymic slices at indicated times after peptide addition with or without zVAD. E) Ratio of total (Aqua^+^ and Aqua^−^ after exclusion of debris and doublets) CD4^+^CD8^+^ F5 and OT I thymocytes recovered from thymic slices at indicated times after peptide addition with or without zVAD. The data in (D) and (E) depict mean with SEM from five compiled experiments done in triplicates and are normalized so that the ratio at time zero without treatment is set to one. (F) Fold increase of Annexin V^+^ CD4^+^CD8^+^ F5 thymocytes with intact membrane recovered from thymic slices at indicated times after peptide addition with or without zVAD. The data depict mean with SEM from two compiled experiments done in triplicates and are normalized so that the ratio at time zero without treatment is set to one.

Having defined the initial events following exposure of thymocytes to negative selecting stimuli *in situ*, we next turned our attention to the endpoints of negative selection, namely cell death and phagocytosis. To do so, we first determined more precisely the time required to complete negative selection using flow cytometric analysis of overlaid thymocytes at various times after peptide addition. To detect apoptotic cells, we used an antibody specific for active caspase 3, an early marker for apoptosis induction, combined with the fixable live/dead dye Aqua, to identify cells that have lost membrane integrity (Fig. 5A). Early apoptotic cells (active caspase 3^+^ Aqua^−^) were detectable above background starting at 2 hours and continuing until 12 hours after peptide addition (Fig. 5B). A similar time course was observed for all apoptotic cells (active caspase 3^+^ Aqua^−^ and active caspase 3^+^ Aqua^+^) (Fig. 5C).

Because dying thymocytes are efficiently cleared by phagocytes, the number of active caspase 3^+^ thymocytes detectable at any given time point provides only a snapshot of negative selection. The cumulative loss of viable thymocytes should provide a more accurate read-out of the extent of negative selection over time, however this measurement is complicated by the variation in the number of thymocytes per slice and seeding of slices by F5 thymocytes. To get around these problems, we included a population of thymocytes bearing an irrelevant TCR (OT I) to serve as internal reference. We overlaid marked OT I and F5 thymocytes on WT thymic slices at a ratio of approximately 1∶1, and determined the number of viable thymocyes of each donor type using flow cytometry at various times after peptide addition. We then used the ratio of F5 to OT I CD4^+^CD8^+^ thymocytes as a measurement of cell loss due to negative selection. With this approach, we were able to detect loss of viable cells as early as 3 hours after the addition of the specific peptide (Fig. 5D), lagging 1 hour after the first appearance of active caspase 3^+^ Aqua^−^ cells (Fig. 5B). By 12 hours the number of non-apoptotic F5 thymocytes had decreased by ∼70% (Fig. 5D). A similar time course was observed when total (Aqua^+^ and Aqua^−^ cells after exclusion of debris and doublets) CD4^+^CD8^+^ thymocytes were quantified (Fig. 5E). PS exposure, which serves as an “eat-me” signal to phagocytes, could be detected above background by 2 hours after peptide addition, corresponding to the first appearance of active caspase 3^+^ cells (Fig. 5F). Importantly, the pan-caspase inhibitor zVAD-fmk (zVAD) abolished both PS exposure and cell loss indicating that caspases are essential for both processes (Fig. 5D, 5E, and 5F). Together these data imply that thymocytes undergo caspase-dependent cell death asynchronously between 3 and 12 hours following synchronous encounter with negative selecting peptide.

### Visualization of the Cell Death and Clearance during Acute Negative Selection

**Figure 6:**
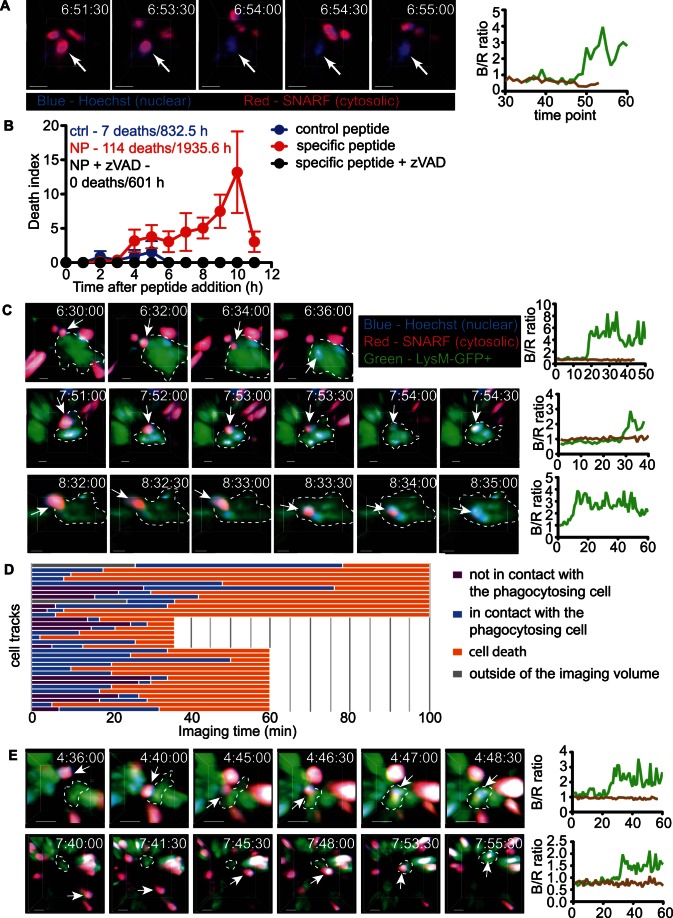
Visualization of cell death and clearance during acute negative selection. (A) Still images from an example of cell death of F5 CD4^+^CD8^+^ thymocyte revealed by SNARF and Hoechst labeling. The dying cell is identified by the arrow. (B) Cell death events over time after treatment with specific peptide with or without zVAD or control peptide. Death index is calculated as number of observed death events per 100 live cells in the first time frame of a movie. Each data point represents mean and SEM from all movies for a given time window. Data are compiled from 31 movies from 2 imaging days for control, 50 movies from three imaging days for specific peptide and 16 movies from one imaging day for specific peptide+zVAD. The inset shows the total number of cell deaths observed and the total cumulative cell imaging hours for each condition. (C) Examples of Hoechst and SNARF fluorescence changes in CD4^+^CD8^+^ F5 thymocytes being engulfed by LysM-GFP phagocytes. (D) Timeline chart depicting the time of contact with the phagocytosing LysM-GFP cells and the time of death (increase of B/R ratio) of 31 tracked F5 CD4^+^CD8^+^ cells. (E) Examples of Hoechst and SNARF labeled CD4^+^CD8^+^ F5 thymocytes that migrate to the phagocytosing LysM-GFP cell. Time after peptide addition is shown in the upper right corner of each snapshot. The arrows in (C) and (E) point to cells that are being phagocytosed. To the right is the ratio of Hoechst to SNARF fluorescence over time (blue to red or B/R ratio) for the dying cell (green) and for a nearby live cell (brown). Scale bars are 5 µm in (A) and (C) and 10 µm in (E).

Having established a time window for negative selection in our system, we next set out to directly visualize thymocyte apoptosis and relate it to phagocyte clearance. To visualize cell death, we adapted a method in which cells are double-labeled with a cytosolic dye (SNARF) to detect the loss of membrane integrity and the nuclear dye Hoechst to detect apoptosis-induced changes in the chromatin [30]. Purified CD4^+^CD8^+^ F5 thymocytes were labeled with SNARF and Hoechst, seeded on thymic slices that were subsequently incubated with specific peptide, and analyzed by time-lapse two-photon microscopy in the medulla. Cell death could be detected by a sudden increase in the ratio of fluorescence in the blue to red channels due to a drop in SNARF and an increase in Hoechst signal (blue to red or B/R ratio) in individual thymocytes (Fig. 6A, white arrow and green line) while the B/R ratio of neighboring thymocytes remained unchanged (Fig. 6A, brown line). While very few examples of cell death were seen during the first 3 hours, numerous examples were seen starting at 4 hours after addition of specific peptide (Fig. 6B and Video S5). Very little cell death was observed with control peptide or in the presence of the caspase inhibitor zVAD (Fig. 6B). Cell death could be readily detected up to 12 hours after addition of specific peptide, the limit for maintaining adequate tissue viability under these conditions. These data are in good agreement with the time course for cell loss detected by flow cytometry (Fig. 5E) and confirm that there is considerable variation in the time to death of individual thymocytes following synchronous encounter with a negative selecting ligand.

To relate thymocyte death to phagocytosis by macrophages, we seeded SNARF and Hoechst labeled CD4^+^CD8^+^ F5 thymocytes onto thymic slices from LysM-GFP reporter mice, in which GFP is expressed by phagocytes that are predominantly located in the medulla (Fig. S1) [31]. In all cases, peptide-induced cell death occurred while the thymocyte was in intimate contact or enclosed by a LysM-GFP^+^ cell (31 out of 31) (Fig. 6C and 6D and Video S5). In contrast, only 54% of viable thymocytes from these same runs were in contact with phagocytes, and only 7% appeared to be in intimate contact or engulfed (n = 1,461). The time between the initial contact with the phagocyte and death varied considerably (2–56 min), however many thymocytes remained tightly associated with phagocytes for the duration of the imaging run (>30 min) while remaining viable (Fig. 6D).

The initial contact between the thymocyte and phagocyte could be observed in ∼45% of all phagocytosis examples (14 out of 31). Surprisingly, in all cases (14 out of 14), it was the slowly moving thymocyte that approached the phagocyte and not the other way around (Fig. 6D and E and Video S6). The majority of LysM-GFP^+^ phagocytes were stationary despite the abundance of dying cells around them, suggesting that, at least in this system, “find-me” signals and directed migration of phagocytes to apoptotic cells are not the prevalent clearance mechanism.

## Discussion

While many of the individual events during thymocyte negative selection have been identified, the relationship between them is unclear, in part because we lack information about the temporal sequence of events as they occur *in vivo*. Here we address this gap in our knowledge by following a synchronized cohort of thymocytes undergoing negative selection from their initial encounter with negative selecting ligands, to their eventual death and phagocytic clearance. We used two-photon time-lapse microscopy to directly visualize thymocyte cell death and phagocytosis, and observed that engulfment by macrophages precedes the permeabilization of the plasma membrane and chromatin condensation. Our work establishes the chronological sequence of events during negative selection, and reveals a surprising close coupling between apoptosis and phagocytosis *in vivo*.

Our experimental setup allowed us to visualize the initial encounter between thymocytes and their negative selecting ligands *in situ*, revealing rapid migratory arrest accompanied by calcium flux. In contrast, autoreactive thymocytes in a steady-state, AIRE-dependent model of negative selection migrated relatively rapidly within confinement zones and no stopping phase was discerned [21]. While AIRE-dependent antigens are thought to be present at relatively low abundance in the thymus [32], it seems unlikely that peptide abundance alone could account for the difference in stopping behavior in the two systems, since we show here that even very low peptide concentrations can induce an all or nothing stopping response in thymocytes. Moreover, a recent report of CD4^+^ SP thymocytes in the presence of an AIRE-dependent negative selecting antigen revealed examples of migratory arrest [33]. TCR signals and migration are interrelated, with signaling inducing migratory arrest, and migratory arrest in turn prolonging contact with a single APC and thus promoting sustained signaling. Thus, thymocytes may tune TCR signaling and migratory arrest to balance between sensitive antigen detection and efficient scanning of multiple thymic APCs. Altogether, these data are consistent with the view that thymocytes undergo calcium signaling and migratory arrest upon initial encounter with a negative selecting ligand, but the subsequent response depends on the intensity of the signal received. Encounter with a high abundance peptide and/or presentation by a more stimulatory APC would lead to continued migratory arrest and cell death within a few hours. On the other hand, in response to encounter with a low abundance peptide and/or presentation by a less stimulatory APC, a thymocyte may recover its motility and continue to sample the thymic environment for some time before either undergoing delayed negative selection, agonist selection [34], or export from the medulla as a mature conventional T cell [35],[36].

In many cases phagocytes migrate toward “find-me” signals released by dying cells [37]. On the other hand, a recent report in a zebra fish model revealed dying neurons actively migrating to regions of the brain containing phagocytes [38]. Here we observe that phagocytes are relatively abundant in the vicinity of dying thymocytes and approximately half of thymocytes are in contact with a phagocyte even in the absence of antigenic peptide. However, in the handful of examples in which we were able to observe the initial encounter between a dying thymocyte and the phagocyte prior to death and engulfment, it was the thymocyte that approached a sessile phagocyte. These observations suggest that a “find-your-phagocyte” model might operate in multiple *in vivo* settings.

Through the use of time-lapse imaging and pharmacological interventions, we were able to elucidate the timing and sequence of events during the executionary phase of apoptosis resulting from negative selection *in vivo*. Caspase activation is the first marker of death induction that appears around 2 hours after pMHC stimulation. Phagocytosis of dying cells is dependent on caspases' enzymatic activity and follows within an hour, possibly mediated by the “eat-me” signal PS. Perhaps the most surprising result from our study is the observation that phagocytosis precedes typical features of apoptosis. The standard morphological description of apoptosis includes nuclear (chromatin) condensation and fragmentation, while the plasma membrane is still intact and cell disaggregation into apoptotic bodies or blebs that are ultimately engulfed by phagocytes [39],[40]. Our *in situ* data suggest that the internal antigens of dying cells are guarded even more strictly than previously appreciated and the phagocytes may engulf the entire cell at the onset of apoptosis before blebbing or nuclear condensation has occurred. Moreover, the observation that chromatin changes are coincident with plasma membrane permeabilization is consistent with the possibility that phagocyte's lysosomal DNases could play a role in the nuclear breakdown. This raises the intriguing possibility that phagocytes may not be merely “undertakers” serving to remove corpses, but may also serve as “executioners” helping to deliver the final deathblow to the autoreactive thymocyte.

A further intriguing possibility is that phagocytes could also serve as antigen presenting cells for negative selection. F4/80^+^ phagocytes can induce negative selection in thymic organ culture, although CD11c^+^ dendritic cells appear to do so more efficiently [41],[42]. Moreover, in situ localization of thymocytes undergoing negative selection to a ubiquitous antigen in the cortex revealed a close association with CD11c^+^ DCs, but not with F4/80^+^ macrophages [14]. The LysM-GFP reporter that we use in this study to identify phagocytes, shows partial overlap with many of the markers used to identify macrophages and DCs, including CD11c (data not shown). The notion that the same cell may participate in both the initiation and the clean up of negative selection awaits further experimental testing.

Previous work has shown that negative selection requires new gene expression [9],[43] and, specifically induction of the pro-apoptotic protein Bim [44]. Thus, the lag period between peptide addition and thymocyte death that we observe could reflect the need to accumulate Bim to levels sufficient to neutralize anti-apoptotic proteins of the Bcl-2 family, and thereby trigger mitochondrial membrane permeabilization, cytochrome C release, and caspase 3 activation. Interestingly, in spite of the fact that all thymocytes responded within minutes to peptide addition, the entry of cells into the death program occurred asynchronously over a period of several hours. Asynchronous cell death has also been reported for cultured cell lines after treatment with a uniform death inducing stimulus [45], a phenomenon attributed to stochastic variation in expression of pro and anti-apoptotic proteins by individual cells [46]. TCR signaling in thymocytes can induce both pro-apoptotic factors such as Bim, as well as pro-survival factors such as Schnurri [47]. Moreover, non-TCR mediated factors such as cytokines may also provide survival signals to autoreactive thymocytes *in vivo*. Thus, it seems likely that autoreactive thymocytes assess the relative levels of various pro and anti-apoptotic factors in choosing when and if to die. Given emerging evidence that some autoreactive thymocytes may escape negative selection and give rise to agonist-selected or conventional peripheral T cells [34],[36], it is tempting to speculate that the prolonged waiting period after encounter with self antigen may allow autoreactive thymocytes to choose between these alternative fates.

## Materials and Methods

### Mice

C57BL/6J (CD45.2), B6.SJL-*Ptprca Pepcb*/BoyJ (CD45.1), Ubi-GFP and Actin-CFP mice were from Jackson Labs. OT I RAG2^−/−^ mice were from Taconic Farms. F5 RAG1^−/−^ and LysM-GFP mice have been described [31],[48]. All the mice were bred and maintained in specific pathogen-free conditions at the animal facility at University of California, Berkeley according to protocols approved by the Institutional Animal Care and Use Committee.

### Chimera Generation

For mixed BM chimera generation, T cell-depleted BM from donor mice (F5 RAG1^−/−^ CD45.2 and C57BL/6J CD45.1, in equal proportions) was injected into the recipients (C57BL/6J CD45.1) irradiated with two doses of 550 rad 4 h apart from a ^147^Cs source. In some experiments, partial hematopoetic chimeras were generated by injecting neonatal C57BL/6J mice with BM from F5 RAG1^−/−^ GFP and Actin-CFP at days 3 and 5 after birth. All chimeras were analyzed after >5 weeks post reconstitution. Peptide injection was carried out with 50 nanomoles of NP_366–374_ peptide (Anaspec) dissolved in PBS, intravenously.

### Thymic Slice Preparation and Culture

Thymic slices were prepared essentially as described [49]. Briefly, thymic lobes cleaned of connective tissue were embedded in 4% GTG-NuSieve Agarose (Lonza) in HBSS and cut with a 1000 Plus sectioning system (Vibratome, Leica) into 0.4 mm thick slices. The slices were laid on 0.4 µm Cell Culture Inserts (BD Biosciences) in 6-well plates (BD Biosciences) that contained 1 ml of complete RPMI (cRPMI) medium and incubated at 37°C in a plastic bag filled with 80% O_2_+15% N_2_+5% CO_2_ (Blood Gas, Praxair). Different amounts of F5 specific, NP_366–374_, and control peptides, Ova_257–264_ or VSV_264–272_ (all from Anaspec) were added in 1 ml of cRPMI and withdrawn after 30 min. In some experiments pan-caspase inhibitor I (zVAD-fmk, EMD) was added to 50 µM final concentration to the medium. In other experiments, the slices were dissociated in 5 ml cRPMI medium, the cell suspension spun down and resuspended in 200 µl cRPMI, and added to 96-well plate for further incubation.

### Thymocyte Purification and Labeling

Thymocyte single cell suspension was prepared in PBS. CD4^+^CD8^+^ F5 thymocytes were depleted of non T cells and mature CD8 SP cells with anti-Biotin MicroBeads and LS columns (Miltenyi Biotech) following incubation with the following biotinylated antibody cocktail – CD11b, CD11c, CD19, CD25, MHC II, DX5, Ter-119, β7-integrin (all from eBioscience or BioLegend). For labeling, 10^7^ thymocytes were incubated with 2 µM Indo-1 LR (Teflabs) at 3.3×10^6^ cells/ml for 90 min in cRPMI at 37°C. In other cases 2×10^7^ thymocytes were labeled with 3 µM SNARF (Invitrogen) at 10^7^ cells/ml for 15 min in pre-warmed PBS at 37°C, washed with cRPMI and further labeled with 5 µM Hoechst 33342 (Invitrogen) at 10^7^ cells/ml for 15 min in pre-warmed cRPMI at 37°C. For overlaying on thymic slices, the cell suspensions were adjusted to 10^6^ cells/20 µl, and 10–20 µl were gently overlaid on slices in Cell Culture Inserts. The cells were left to migrate into the slice for 2 h at 37°C/5% CO_2_ and then the cells that had failed to enter the slice were removed by gently washing with PBS.

### Flow Cytometry

Single cell thymocyte suspensions were blocked with 24G2 supernatant for 10 min in ice and 2×10^6^ cells were stained with antibodies for surface markers (CD4, CD8, CD44, CD45.1, CD45.2, CD69, TCRβ in Pacific Blue, FITC, PerCP/cy5.5. PE/cy7, APC, and APC/eFluor780, all from eBioscience or BioLegend) in 0.5% BSA in PBS (FACS buffer). For PS exposure, the cells were washed with DPBS and stained with Annexin V-AlexaFluor 647 (Invitrogen) in binding buffer for 15 min at room temperature in the dark followed immediately by fixation. For Live/Dead fixable Aqua (Invitrogen) staining (or the equivalent fixable viability dye eFluor 506 from eBioscience) the cells were washed with PBS and stained in 100 µl PBS with 1∶500 dilution from the dyes for 30 min in ice. The cells were washed with PBS and analyzed by flow cytometry or fixed with 2% paraformaldehyde (Electron Microscope Sciences) in PBS for 20 min in ice. For active caspase 3 intracellular staining, the fixed cells were permeabilized/blocked in 0.1% Saponin (Sigma) in FACS buffer+5% normal donkey serum (Jackson Immunoresearch)+10% 24G2 supernatant for 20 min in ice. Anti-active caspase 3 antibody (Cell Signaling) was applied for 40 min at 1∶400 and was detected with anti-rabbit PE (Jackson Immunoresearch). Flow cytometry was performed on LSR II or Fortessa (BD Biosciences) and data analysis was carried out with FlowJo (TreeStar).

### Two-Photon Microscopy

Thymic slices or intact thymic lobes were glued on coverslips and imaged by two-photon laser scanning microscopy with a custom-built up-right microscope or Zeiss 7 MP (Zeiss), while being perfused with warmed (37°C), oxygenated phenol-free DMEM medium (GIBCO) at a rate 1 ml/min. Mode-locked Ti:sapphire laser Mai-Tai (Spectra-Physics) or Chameleon (Coherent) was tuned to 900 nm for CFP+GFP and Hoechst+SNARF excitation or 720 nm for Indo-1 LR excitation with appropriate filter sets. Imaging volumes of various sizes were scanned every 30 sec for 20–60 min and assigned to cortex or medulla based on distance from the capsule (detected by second harmonic signal), density of LysM-GFP cells (greater in the medulla) and the characteristic lower position of the medullary region. Most of the imaging was done in the medulla, except where stated otherwise, because of the superior image quality. Perfusion with peptides was achieved by switching the perfusion medium to phenol-free DMEM containing various amounts of specific or control peptides. Due to the dead volume of the tubing it took 2–3 min for the peptide to reach the sample.

### Image Analysis

Imaris 7.3 (Bitplane) was used to determine cell positions over time and tracking. The x, y, and z coordinates as well as the mean fluorescence intensities of the tracking spots for Ca^2+^-bound Indo-1 LR, Ca^2+^-free Indo-1 LR, Hoechst and SNARF were exported. Motility parameters were calculated in MATLAB (Mathworks) with a custom code that is available upon request. Interval speed is calculated by dividing displacement over time and the time interval is denoted. For example, when adjacent time points were used for the speed calculation it was denoted as “Interval speed (30 sec)” or when it was calculated over five time points it was denoted as “Interval speed (150 sec)”. Ca^2+^-ratio was calculated as a surrogate for Ca^2+^ intracellular concentration by dividing the mean fluorescence intensity of Ca^2+^-bound Indo-1 LR by the Ca^2+^-free Indo-1 LR with great care taken to avoid saturation of pixels. All the values were normalized so that the average Ca^2+^-ratio before peptide addition was zero (corrected Ca^2+^-ratio). Blue/Red fluorescence (B/R) ratio as a measurement for the cell viability was calculated by dividing the mean fluorescence intensity of Hoechst (blue) by SNARF (red).

### Statistical Analysis

Prism 5.0 (GraphPad) was used for graphing and statistical analysis. Unpaired two-tailed t-test was used to determine significance when comparing between two groups, and one-way ANOVA with Tukey post-test was applied when more than two groups were compared.

## Supporting Information

Figure S1 Distribution of LysM-GFP^+^ phagocytes in the cortical and medullary regions of the thymus. Two-photon microscopy image of a thymic slice from a LysM-GFP^+^ mouse overlaid with SNARF+Hoechst labeled purified CD4^+^CD8^+^ F5 transgenic thymocytes. The image is a maximal intensity projection of adjacent three-dimensional imaging volumes spanning the slice from the dorsal to the ventral side. Scale bar, 100 µm. The data are representative of more than 15 independent experiments.
(TIF)

Video S1 Example of thymocytes undergoing migratory arrest upon addition of specific peptide to the perfusion media. Thymus slices from BM chimeras containing GFP-expressing F5 thymocytes (green) and CFP-expressing WT thymocytes (blue) were imaged by time-lapse two-photon microscopy and peptide specific for F5 was added during the imaging run. The trajectories of selected cells are represented as tracks that are colored cyan for F5 and magenta for WT thymocytes. The duration of the movie is 30 min, frames were taken 30 sec apart, peptide was added at ∼13.5 min, the scale bar is 30 µm.
(MOV)

Video S2 A second example of thymocytes undergoing migratory arrest upon addition of specific peptide to the perfusion media. Thymic slices from WT mice were overlaid with F5 (labeled with SNARF – red) and OT I (labeled with CFSE – green) CD4^+^CD8^+^ thymocytes. After 2 hours to allow for thymcoytes to migrate into the slice, samples were imaged by time-lapse two-photon microscopy. Peptide specific for F5 thymocytes was added during the imaging run. The trajectories of selected cells are represented as tracks that are colored magenta for F5 (antigen-specific) and cyan for OT I (irrelevant specificity control) thymocytes. The duration of the movie is 30 min, the scale bar is 50 µm.
(MOV)

Video S3 Calcium flux and migratory arrest of F5 thymocytes after treatment with 1 µM of specific peptide. Indo-1 LR labeled purified CD4^+^CD8^+^ F5 thymocytes were introduced into thymic slices from WT mice and imaged by time-lapse two-photon microscopy. Peptide specific for F5 thymocytes was added during the imaging run. The trajectories of selected cells are represented as white tracks. The graph to the right, in the second movie, shows corrected Ca^2+^-ratio (red line) and the interval speed (150 sec) (blue line) for an individual thymocyte over time. The arrow shows the time of peptide addition. The scale bar is 10 µm.
(MOV)

Video S4 Calcium flux and migratory arrest of F5 thymocytes after treatment with a 100 pM of specific peptide. Indo-1 LR labeled purified CD4^+^CD8^+^ F5 thymocytes were introduced into thymic slices from WT mice and imaged by time-lapse two-photon microscopy. Peptide specific for F5 thymocytes was added during the imaging run. The trajectories of selected cells are represented as white tracks. The graph to the right, in the second movie, shows corrected Ca^2+^-ratio (red line) and the interval speed (150 sec) (blue line) for an individual thymocyte over time. The arrow shows the time of peptide addition. The scale bar is 20 µm.
(MOV)

Video S5 Examples of cell death *in situ* during negative selection. Purified CD4^+^CD8^+^ F5 thymocytes labeled with SNARF and Hoechst were introduced into LysM-GFP thymic slices then treated with 1 nM specific peptide for 30 min. The incubation was continued for the indicated times and the slices were imaged by time-lapse two-photon microscopy. The arrowheads point to dying thymocytes. Scale bars are 5 µm.
(MOV)

Video S6 Examples of thymocytes migrating to phagocytes during negative selection. Purified CD4^+^CD8^+^ F5 thymocytes labeled with SNARF and Hoechst and introduced into LysM-GFP thymic slices, then treated with 1 nM specific peptide for 30 min. The incubation was continued for various times and the slices were imaged by time-lapse two-photon microscopy. Scale bars are 10 µm.
(MOV)

## Abbreviations

APC: antigen-presenting cell
BM: bone marrow
CFP: cyan fluorescent protein
DC: dendritic cell
DP: CD4^+^CD8^+^ double positive
GFP: green fluorescent protein
MHC: major histocompatibility complex
PS: phosphatidylserine
SP: CD4^+^CD8^−^ or CD4^−^CD8^+^ single positive
TCR: T cell antigen receptor
WT: wild type
