# Supplementary figures and images for: Elimination of Self-Reactive T Cells in the Thymus: A Timeline for Negative Selection
Source: PLoS Biol. 2013 May 21;11(5):e1001566. doi: 10.1371/journal.pbio.1001566 (PMC3660248; doi:10.1371/journal.pbio.1001566)

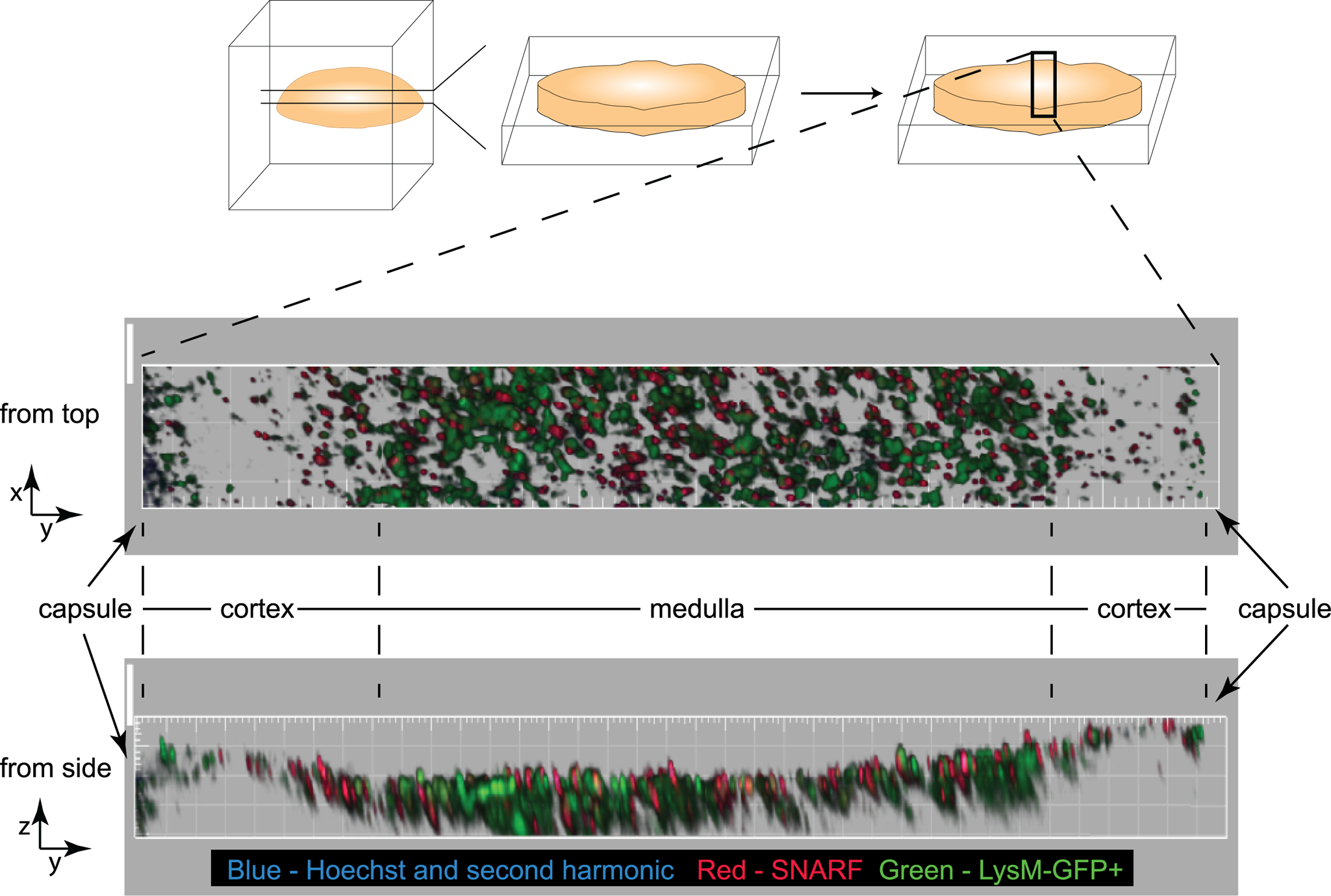

Supplement: Figure S1 — Distribution of LysM-GFP+ phagocytes in the cortical and medullary regions of the thymus. Two-photon microscopy image of a thymic slice from a LysM-GFP+ mouse overlaid with SNARF+Hoechst labeled purified CD4+CD8+ F5 transgenic thymocytes. The image is a maximal intensity projection of adjacent three-dimensional imaging volumes spanning the slice from the dorsal to the ventral side. Scale bar, 100 µm. The data are representative of more than 15 independent experiments. (TIF) [file pbio.1001566.s001.tif]
